# Frontlines burning: Women’s lives at the heart of climate injustice

**DOI:** 10.1371/journal.pgph.0005801

**Published:** 2026-02-20

**Authors:** Shubha Nagesh, Monalisha Sahu

**Affiliations:** 1 Independent Consultant, Dehradun, India; 2 Associate Professor & Head, Dept of Occupational Health, All India Institute of Hygiene & Public Health, Kolkata, India; PLOS: Public Library of Science, UNITED STATES OF AMERICA

“Climate change is not gender neutral. It magnifies the inequalities that already exist.” UN Women, Gender Equality in Climate Action [1]

The climate crisis is an escalating emergency compounded by widening inequalities [2]. In the current context of diminishing global health investments and challenged gender equity movements, climate change acts as a critical multiplier, exacerbating pre-existing social and structural vulnerabilities [2].

In climate-related disasters, women and children are 14 times more likely to die than men—not because of biology, but because of systemic barriers: limited access to early warnings, lower social mobility, and restrictive gender roles [3]. Women also make up nearly 80% of those displaced by climate impacts, yet they remain largely invisible in adaptation plans [4].

Climate change is a major threat multiplier for women’s health—driving heat-related pregnancy risks, worsening conditions like PCOS and anemia, and intensifying exposure to indoor air pollution in low-income and rural settings. Rising temperatures, water scarcity, and climate displacement also erode menstrual hygiene, deepen period poverty, and heighten anxiety and depression among women who shoulder primary household and survival responsibilities [5–7].

Climate-induced crises push girls out of school first—girls are 2.5 times more likely than boys to stop attending—fueling early marriage and lifelong poverty, even though keeping girls in school is one of the strongest and most cost-effective tools for climate resilience, a lever now threatened by shrinking education budgets [8].

Women make up over 60% of the world’s informal workforce—often in climate-exposed sectors like agriculture, fisheries, and domestic work—yet they lack basic safety nets, and in countries like India, where women are nearly 60% of the agricultural workforce but own only 13% of the land, this vulnerability becomes both structural and generational [9,10] Fig 1.

**Fig 1:**
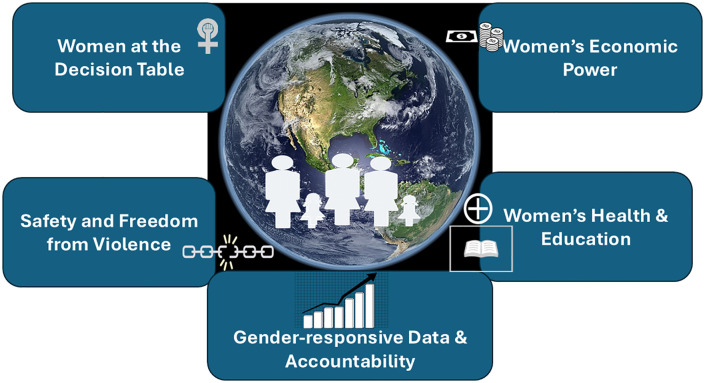
Pictorial Depiction for a feminist climate agenda demands action against Five potential domains.

Gender-based violence can rise by up to 30% after climate disasters, yet fewer than 15% of National Adaptation Plans even mention GBV prevention—leaving women and girls at heightened risk as extreme weather erodes privacy, weakens protection systems, and turns basic spaces like toilets into sites of fear, a predictable consequence of failing to integrate gender into climate response [11].

Women are on the frontlines of the climate crisis but remain largely excluded from decision-making, with fewer than 34% of UNFCCC delegates being women and even fewer holding real power. This exclusion creates a justice gap: when women aren’t at the table, issues like menstrual health, unpaid care work, and gender-based violence are ignored, reflected in the fact that only 15% of national climate plans mention gender at all [12].

At COP30 in Belém, Brazil, the gender agenda has once again highlighted deep inequities: rich nations continue to drag their feet on funding, while women—especially Indigenous and rural leaders—demand meaningful inclusion in decision-making. COP negotiators have proposed a Gender Action Plan, but weakening language, political pushback, and financing shortfalls risk undermining its impact. Meanwhile, in the current geopolitical climate of growing insecurity, shrinking climate aid, and cuts to women’s rights, this summit shows how fragile progress can be when gender justice remains more of a promise than a priority [13].

Feminist climate justice is more than symbolic inclusion it demands breaking down the power structures that silence women’s voices. It makes clear that true climate resilience depends on equity, recognizing menstrual health as climate health, care work as climate work, and representation as essential climate action [14]. Evidence shows that countries with more women in parliament are likelier to ratify environmental treaties, adopt stronger climate policies, and invest more in health and education [15]. When women lead, climate action is fairer, stronger, and more effective. A feminist just transition goes beyond adjusting policies; it demands a complete system transformation. It calls for:

- Fair redistribution of resources like land, funding, and clean energy,
- Official recognition of women’s unpaid work and its inclusion in economic planning,
- Equal opportunities for women to access technology, develop skills, and participate in decision making. Because without sharing power, resilience will stay a privilege for few, not a right for all.

## The way forward: A blueprint for gender-responsive climate action

Addressing climate change without tackling gender inequality is like trying to treat a burn without extinguishing the fire. To build climate resilience that truly protects all, we must centre women’s voices and experiences—not as beneficiaries, but as architects of the future. A feminist climate agenda demands (Fig 1):

- Including Women at the Decision Table: From community action to global negotiations—women must help shape the policies and solutions that affect their lives.
- Increasing Women’s Economic Power: Secure land rights, expand access to climate—resilient jobs, credit, and green tools so women can lead in the transition.
- Funding Women’s Health and Education: Build climate—ready health systems, ensure menstrual health access, and keep girls learning—even during emergencies.
- Tracking Impact by Gender: Collect and use gender—disaggregated data to understand who’s being hit hardest—and tailor responses accordingly.
- Ending Gender—Based Violence in Climate Disasters: Design safe shelters, train responders on GBV prevention, and fund mental health and trauma support.
- Embedding Gender in National Climate Plans: Gender equity must be a core target—not a tick box or afterthought—in national climate commitments.
- Trusting Local, Women—Led Solutions: Fund and support Indigenous women, local cooperatives, and grassroots leaders who are already adapting and innovating on the frontlines.

## Conclusion

Climate change is exposing the cracks in our systems—and gender inequality runs through them all. The truth is simple: we can’t build climate resilience on unequal ground. If women and marginalized communities continue to bear the brunt of the crisis without a seat at the decision—making table, our solutions will be incomplete—and our progress, fragile. Gender justice isn’t a side issue. It’s central to how we adapt, survive, and thrive in a changing world. Because real climate action means shifting power, centering care, and rebuilding systems that work for everyone—not just a few. Without equity, there is no resilience. And without justice, there is no future.
